# Glutamyl aminopeptidase in microvesicular and exosomal fractions of urine is related with renal dysfunction in cisplatin-treated rats

**DOI:** 10.1371/journal.pone.0175462

**Published:** 2017-04-11

**Authors:** Andrés Quesada, Ana Belén Segarra, Sebastián Montoro-Molina, María del Carmen de Gracia, Antonio Osuna, Francisco O’Valle, Manuel Gómez-Guzmán, Félix Vargas, Rosemary Wangensteen

**Affiliations:** 1 Unidad de Nefrología, Hospital Virgen de las Nieves, FIBAO, Granada, Spain; 2 Área de Fisiología, Departamento de Ciencias de la Salud, Universidad de Jaén, Jaén, Spain; 3 Departamento de Anatomía Patológica, IBIMER, Universidad de Granada, Granada, Spain; 4 Departamento de Fisiología, Facultad de Medicina, Universidad de Granada, Granada, Spain; National Institutes of Health, UNITED STATES

## Abstract

**Purpose:**

The aim of this work was to investigate if the content of glutamyl aminopeptidase (GluAp) in microvesicular and exosomal fractions of urine is related with renal dysfunction in cisplatin-treated rats.

**Methods:**

Urine samples were collected 24 hours after injection of cisplatin (7 mg/kg, n = 10) or saline serum (n = 10), and they were subjected to differential centrifugation at 1.000, 17.000 and 200.000 *g* to obtain microvesicular and exosomal fractions. GluAp was measured with a commercial ELISA kit in both fractions. Serum creatinine (SCr) and body weight were measured 15 days after treatment. We analyzed if early excretion of GluAp in microsomal and exosomal fractions was correlated with final SCr and body weight increase. In a second experiment, enzymatic activities of GluAp and alanyl aminopeptidase (AlaAp) in urine, microvesicular and exosomal fractions were measured three days after injection. We analyzed the correlation of both markers with SCr determined at this point. Finally, we studied the expression of GluAp and extracellular vesicles markers Alix and tumor susceptibility gene (TSG101) in both fractions by immunoblotting.

**Results:**

GluAp excretion was increased in all fractions of urine after cisplatin treatment, even if data were normalized per mg of creatinine, per body weight or per total protein content of each fraction. We found significant predictive correlations with SCr concentration, and inverse correlations with body weight increase determined 15 days later. Three days after injection, aminopeptidasic activities were markedly increased in all fractions of urine in cisplatin-treated rats. The highest correlation coefficient with SCr was found for GluAp in microvesicular fraction. Increase of GluAp in microvesicular and exosomal fractions from cisplatin-treated rats was confirmed by immunoblotting. Alix and TSG101 showed different patterns of expression in each fraction.

**Conclusions:**

Determination of GluAp content or its enzymatic activity in microvesicular and exosomal fractions of urine is an early and predictive biomarker of renal dysfunction in cisplatin-induced nephrotoxicity. Measurement of GluAp in these fractions can serve to detect proximal tubular damage independently of glomerular filtration status.

## Introduction

Early diagnosis of acute kidney injury remains to be a challenge nowadays. Traditional biomarkers, like serum blood urea nitrogen (BUN) or creatinine (SCr) start to rise when kidney has lost at least a 50% of its function [1,2] and they have been considered by some authors as delayed biomarkers [3].

Consequently, several biomarkers such as N-acetyl-β-D-glucosaminidase (NAG), kidney injury molecule-1 (KIM-1) or neutrophil gelatinase-associated lipocalin (NGAL) have been proposed during the last years as early markers of renal dysfunction in different pathologies, and urine is the most promising source of substances that can be related with the extent of kidney injury [4].

Cisplatin is an antineoplasic drug that induces nephrotoxicity as a collateral effect [5,6], and it has been widely used to evoke kidney injury in experimental models [7]. In previous works, we have demonstrated that urinary activities of aminopeptidasic enzymes, mainly alanyl (Ala) and glutamyl aminopeptidase (GluAp) are early increased in cisplatin-treated rats. [8,9].

Urine can contain extracellular vesicles (EVs) that are released from renal epithelium [10,11]. Although there is not a general consensus for classification criteria and isolation methods for the different kinds of EVs, differential centrifugation is the most commonly used method to isolate EVs from biofluids [10]. These EVs include exosomes, that are nanovesicles (30–120 nm) secreted by epithelial cells of urinary tract [12,13], and ectosomes, also referred as microvesicles (MVs), that are bigger (100–1000 nm) than exosomes and they are produced by the direct budding of the plasma membrane [13]. Amount and composition of exosomes excreted in urine might be important in diagnosis of renal dysfunction, because they can constitute a noninvasive source of multiple disease biomarkers that could provide clinically useful information [14,15,16].

GluAp was identified in urinary exosomes using proteomic analysis [17], but the presence of this enzyme in microvesicles has not been studied. With this background, the aim of this work was to study if GluAp could be quantified in exosomal and microsomal fractions obtained by differential centrifugation of urine, and to analyze if the early excretion of this enzyme in the different fractions was related with renal dysfunction in cisplatin-treated rats.

## Materials and methods

### Experimental design

Twenty male Wistar rats with a weight from 175 to 234 g were purchased from Harlan Laboratories (Barcelona, Spain) and distributed in two groups: control and cisplatin (n = 10 each group). Cisplatin (Sigma-Aldrich, Madrid, Spain) was dissolved in sterile saline solution (3.5 mg/ml). Animals of Cisplatin group were subcutaneously injected with 2 ml/kg of this solution, receiving a dose of 7 mg/kg of cisplatin, while animals of Control group were injected with 2 ml/kg of sterile saline solution. Urine samples were taken in a metabolic cage during the next 24 h. 15 days after injection, blood samples were obtained from left ventricle under anesthesia (pentobarbital, 50 mg/kg i.p.), centrifugated at 1000 *g* during 15 minutes at 4°C, and frozen at -80°C until analysis.

In a second experiment, twenty male Wistar rats purchased from Envigo (Barcelona, Spain) weighing from 360 to 469 g were distributed in Control and Cisplatin groups (n = 10 each group). Cisplatin group was injected with 7 mg/kg of cisplatin and Control group was injected with saline serum as described before. Urine and blood samples were collected three days after treatment, at the peak of cisplatin nephrotoxicity, and processed as described.

### Ethics statement

All experimental procedures were performed in strict accordance with the European Union Guidelines to the Care and Use of Laboratory Animals. This study was approved by the Ethical Committee of the Universidad de Jaén and Junta de Andalucía with the approval number 450–5297. All surgery was performed under sodium pentobarbital anesthesia, and all efforts were made to minimize suffering.

### Processing of urine samples

Isolation method of microsomal and exosomal fractions from urine was modified from Zhou et al. [18]. Urine samples were collected 24 hours after injection and centrifugated at 1000 *g*, 10 minutes, 4°C in order to separate whole cells, bacteria, cellular debris and other substances present in urine. Precipitates were discarded. Some aliquots of supernatants were frozen at -80°C to determine creatinine. The rest of supernatant was treated with protease inhibitors. 1 μl of 1 mM leupeptin and 50 μl of 10 mM phenylmethylsulfonyl fluoride (PMSF) were added to each ml of supernatant.

Supernatants containing protease inhibitors were subjected to a second centrifugation step at 17000 *g*, 15 minutes, 4°C. Precipitates including microsomal fraction (microvesicles, ectosomes, large membrane fragments) were redissolved in 0.5 ml of 20 mM HCl-Tris, pH 8.6 and frozen at -80°C until processing.

Finally, supernatants were subjected to ultracentrifugation at 200000 *g*, 1 hour, 4°C. Precipitates containing exosomal fraction were redissolved in 0.3 ml of 20 mM HCl-Tris, pH 8.6, and frozen at -80°C.

In the second experiment, urine samples were collected three days after injection and processed as described. Protease inhibitors were not added in order to determine GluAp and AlaAp enzymatic activities in supernatant, microvesicular and exosomal fractions.

### Measurement of GluAp content

GluAp was measured in microsomal and exosomal fractions obtained from urine 24 hours after injection as previously described, using an ELISA kit from Sunred Biotechnologies (Shanghai, China). All samples and standards were measured in duplicate. Absorbances of all samples were inside the range of the standard curve (from 4 to 64 ng/ml). Concentration of GluAp in microsomal and exosomal fractions was divided by the corresponding factor and referred to the starting volume of urine.

### Measurement of GluAp and AlaAp activities

GluAp and AlaAp fluorimetric activities were determined three days after injection in a kinetic fluorimetric assay using L-glutamic acid γ-2-naphthylamide or alanyl-2-naphthylamide (Sigma-Aldrich, Madrid, Spain) as substrates, respectively. 20 μl of urine were incubated during 30 min at 37°C with 80 μl of substrate solution (10 mM L-glutamic acid γ-2-naphthylamide or 10 mM alanyl-2-naphthylamide in pH 8.7 50 mM HCl-Tris). Substrates had been previously dissolved in 1 ml of dimethyl sulfoxide and stored at -20°C. The amount of 2-naphthylamine released as a result of the aminopeptidase activities was measured fluorimetrically at an emission wavelength of 412 nm with an excitation wavelength of 345 nm, and quantified using a standard curve of 2-naphthylamine (0–200 nmol/ml). Fluorimetric data from samples and standard curve were taken each minute. Specific aminopeptidase activities were calculated from the slope of the linear portion of enzymatic assay, and expressed as nanomol of substrate hydrolyzed per minute per mg of urine creatinine (mU).

### Measurement of creatinine and protein content

Creatinine and proteinuria was measured in supernatants from all samples of urine in a Spin120 autoanalyzer using reagents purchased from Spinreact (Barcelona, Spain).

Protein concentration in microsomal and exosomal fractions was measured in a 96-well microplate using a DC Protein Bioassay kit from Biorad Laboratories, Madrid, Spain.

### Immunoblotting studies

50 ml of urine samples from control rats and 200 ml from cisplatin-treated rats obtained 3 days after treatment with cisplatin were pooled and subjected to the two-steps centrifugation at 17.000 and 200.000 g. Pellets were dissolved in 50 mM Tris-HCl (pH 6.8) containing 1.5% SDS. Total protein content was measured with DC Protein Bioassay kit from Biorad Laboratories, Madrid, Spain. 30 μg of protein were subjected to SDS-PAGE, transferred to a nitrocellulose membrane, blocked with 1% casein (Bio-Rad Laboratories) and probed overnight at 4°C with 1μg/ml of goat anti-GluAp-antibody (Everest Biotech, Upper Heyford, UK), 1 μg/ml of rabbit anti-Alix-antibody (Sigma-Aldrich, Madrid, Spain) or 1 μg/ml of rabbit anti-TSG101-antibody (Sigma-Aldrich, Madrid, Spain). After washing, membranes were probed with 0.04 μg/ml rabbit anti-goat horseradish peroxidase-linked IgG antibody (KPL Inc., Gaithersburg, MD, USA) or 0.04 μg/ml mouse anti-rabbit horseradish peroxidase-linked IgG antibody (KPL Inc., Gaithersburg, MD, USA) as secondary antibodies. Bands were visualized with ECL (Amersham, Amersham, UK) in a CCD camera image system.

### Statistical analysis

We used t test for the analysis of variables with normal distribution and equal variances, Welch modification of t test was used for data with normal distribution and unequal variances and Mann–Whitney W (Wilcoxon) test was used to analyze the differences when data did not correspond to a normal distribution. Shapiro–Wilk test was used to analyze the normality of distributions. Differences were considered statistically significant at p < 0.05 level.

Linear regressions were made with StatGraphics software to establish the correlation of GluAp or AlaAp in the different fractions with SCr and body weight increase.

## Results and discussion

### Cisplatin increased microvesicular and exosomal content of GluAp

**Fig 1:**
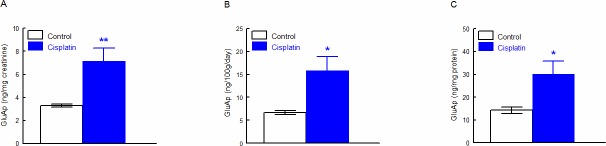
GluAp in microvesicular fraction. GluAp excretion in microvesicular fraction of urine samples from Control and Cisplatin groups collected 24 hours after injection. Data were expressed in ng/mg creatinine (A), ng/100g/day (B) and ng/mg protein (C). Mean ± SEM; * p<0.05, ** p<0.01 Cisplatin *vs*. Control (n = 10 each group).

**Fig 2:**
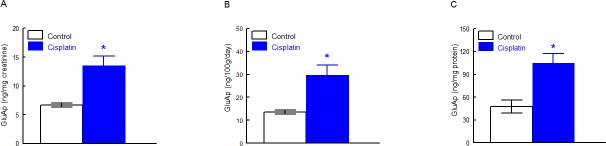
GluAp in exosomal fraction. GluAp excretion in exosomal fraction of urine samples from Control and Cisplatin groups collected 24 hours after injection. Data were expressed in ng/mg creatinine (A), ng/100g/day (B) and ng/mg protein (C). Mean ± SEM; * p<0.01 Cisplatin *vs*. Control (n = 10 each group).

Cisplatin treatment induced a significant increase in GluAp excreted in microvesicular (Fig 1) and exosomal (Fig 2) fractions of urine 24 hours after injection. Interestingly, excretion of GluAp was found to be increased in both fractions of urine when data were expressed in ng/mg creatinine, ng/100g/day and ng/mg of protein content in each fraction.

Urinary markers are usually normalized per mg of urine creatinine, useful for spot samples, or per daily total excretion when data of diuresis are available. Nevertheless, alterations in glomerular filtration are accompanied with a low excretion of urinary creatinine that could falsely increase the levels of urinary markers. Besides, alterations in diuresis can also influence the quantification of urinary markers, because differences in water content can affect biochemical determinations. In our study, cisplatin induced an increase in microvesicular and exosomal GluAp content normalized per mg of total protein in each fraction, implicating that nephrotoxic effect of cisplatin over tubular epithelia could be assessed independently of creatinine status or diuresis.

Besides, augmentation in GluAp excretion were not influenced in a high extent by creatinine excretion or total protein content in each fraction, because there was not any difference in these variables between both groups of rats, although diuresis was significantly higher in cisplatin-treated rats (Table 1).

**Table 1:** Urinary variables 24 hours after injection.

	Control	Cisplatin
UCr (mg/100g/day)	2.03 ± 0.07	2.15 ± 0.12
Microvesicular protein (μg/mg crea)	250 ± 25.5	246 ± 9.56
Exosomal protein (μg/mg crea)	191 ± 36.4	130 ± 7.66
Diuresis (ml/100g)	6.94 ± 0.52	16.8 ± 2.47*

Urine creatinine excretion (UCr; mg/100g/day), microvesicular (μg/mg creatinine) and exosomal (μg/mg creatinine) protein content, and diuresis (ml/100g) in Control and Cisplatin groups 24 hours after injection. Data are expressed as mean ± SEM.

* p<0.01 Cisplatin *vs* Control (n = 10 each group).

The high content of GluAp in exosomal and microsomal fractions that we have found in this study could be due to a direct effect of cisplatin over proximal tubule that would evoke a higher release of microvesicles and exosomes from this section of nephron where aminopeptidasic enzymes are mainly expressed [19,20]. This could explain that microsomal and exosomal fractions were richer in GluAp content in cisplatin-treated rats than in control group. In this way, other authors have stated that exosomes may be the best source of biomarkers for renal tubulopathies, *i*.*e*. disorders that affect the function of renal tubule epithelia [12].

### Microvesicular and exosomal content of GluAp was related with renal dysfunction

**Fig 3:**
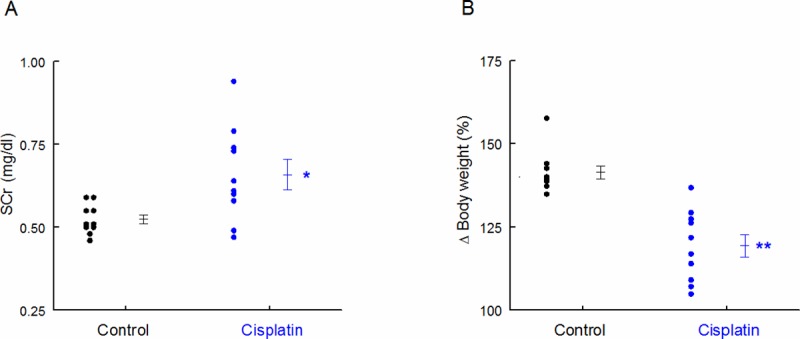
SCr and body weight. SCr concentration (mg/dl) (A) and body weight increase (%) in Control and Cisplatin groups at the end of the experiment. Individual samples and means ± SEM are displayed; * p<0.05, **p<0.001 Cisplatin *vs*. Control (n = 10 each group).

Two weeks after injection, SCr concentration remained significantly increased in cisplatin-treated rats *vs* control group, and body weight was decreased (Fig 3). We found significant correlations between microvesicular and exosomal GluAp excreted 24 hours after cisplatin treatment and both parameters of renal damage-associated morbidity at the end of the experiment.

**Fig 4:**
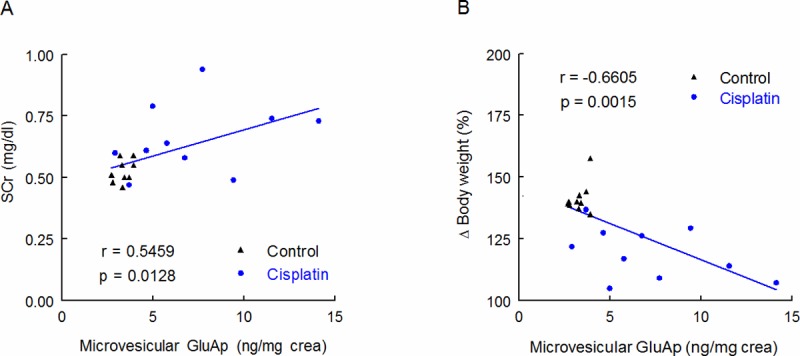
Correlations with microvesicular fraction. Linear regressions between GluAp excretion (ng/mg creatinine) in microvesicular fraction of urine samples collected 24 hours after injection with SCr concentration (mg/dl) (A) and body weight increase (%) (B) determined at the end of the experiment.

GluAp content in microvesicular fraction was significantly correlated with SCr and negatively correlated with body weight increase (Fig 4), indicating that the determination of this enzyme in microvesicles can also be an early marker of kidney injury.

**Fig 5:**
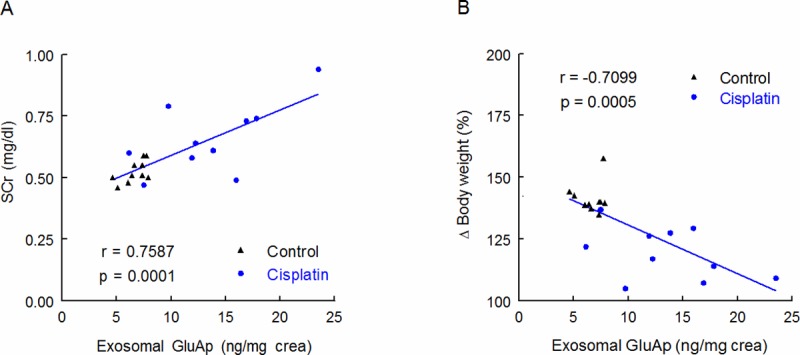
Correlations with exosomal fraction. Linear regressions between GluAp excretion (ng/mg creatinine) in exosomal fraction of urine samples collected 24 hours after injection with SCr concentration (mg/dl) (A) and body weight increase (%) (B) determined at the end of the experiment.

Exosomal GluAp (ng/mg creatinine) showed a very strong correlation with SCr and body weight decrease (Fig 5). Correlation coefficient and *p*-value were higher than those obtained for soluble GluAp in a previous work [9], and they were also higher than those obtained for microvesicular GluAp (Fig 4 and Table 2) or exosomal GluAp expressed in ng/100g/day or ng/mg of protein (Table 3). Therefore, in our study, quantification of exosomal GluAp in ng/mg of creatinine was more related with renal dysfunction, probably because slight alterations in creatinine excretion can also contribute to later renal dysfunction. But correlation of exosomal GluAp expressed in ng/mg protein was also very strong (Table 3). Thus, it might also be a very useful marker in pathologies where evaluation of early tubular damage takes relevance.

**Table 2:** Correlations with microvesicular fraction.

	r	p
*GluAp (ng/100g/day)*		
SCr (mg/dl)	0.4661	0.0383
Δ Body weight (%)	-0.5882	0.0064
*GluAp (ng/mg prot)*		
SCr (mg/dl)	0.4680	0.0374
Δ Body weight (%)	-0.6275	0.0031

Correlation coefficient (r) and statistical significance (p) of linear regressions between GluAp excretion expressed in ng/100 g/day and ng/mg protein in microvesicular fraction of urine samples collected 24 hours after injection, with SCr concentration and body weight increase determined at the end of the experiment.

**Table 3:** Correlations with exosomal fraction.

	r	p
*GluAp (ng/100g/day)*		
SCr (mg/dl)	0.6472	0.0020
Δ Body weight (%)	-0.6264	0.0031
*GluAp (ng/mg prot)*		
SCr (mg/dl)	0.6982	0.0006
Δ Body weight (%)	-0.6887	0.0008

Correlation coefficient (r) and statistical significance (p) of linear regressions between GluAp excretion expressed in ng/100 g/day and ng/mg protein in exosomal fraction of urine samples collected 24 hours after injection, with SCr concentration and body weight increase determined at the end of the experiment.

In previous works, we found an early high increase in the excretion [9] or activity [8] of this enzyme in supernatant of urine from cisplatin-treated rats that were correlated with the extent of renal damage. Our previous studies also demonstrated that the increased excretion of GluAp was earlier than the excretion of other renal markers like NAG or NGAL [8]. Measurement of GluAp in exosomal and microsomal fractions can suppose a technical improvement with respect to analysis of urine, because samples are subjected to two centrifugation steps and precipitates are dissolved in a buffer [18]. Therefore, interferences in the determination of biomarkers that can be evoked by the urea content, ionic strength or other components of urine are avoided. The high correlation that we found between the excretion of the enzyme in these urinary fractions with the augmentation in SCr concentration or body weight decrease can also constitute a useful tool to classify different levels of renal dysfunction.

### GluAp and AlaAp activities were highly increased in urine, microvesicular and exosomal fractions at the peak of toxicity

GluAp activity was highly increased three days after injection in urine supernatant, microvesicular and exosomal fractions from cisplatin-treated rats when data were normalized by urinary creatinine, body weight or total protein content of each fraction, and it was even increased per ml of urine (Table 4). This latter finding implicates that excretion of this marker is augmented independently of diuresis, creatinine concentration, body weight or protein content in all analyzed fractions.

**Table 4:** GluAp activity in urine fractions.

	Control	Cisplatin	Relative increase
*Supernatant*			
GluAp (mU/ml)	0.58 ± 0.09	2.35 ± 0.26**	4.07
GluAp (mU/mg creatinine)	1.11 ± 0.22	18.9 ± 2.05**	17.0
GluAp (mU/100g/day)	2.45 ± 0.54	22.4 ± 2.84**	9.13
GluAp (mU/mg protein)	1.39 ± 0.28	2.61 ± 0.15*	1.88
*Microsomal fraction*			
GluAp (mU/ml)	0.23 ± 0.05	0.94 ± 0.07**	4.12
GluAp (mU/mg creatinine)	0.42 ± 0.09	8.23 ± 1.27**	19.6
GluAp (mU/100g/day)	0.92 ± 0.21	9.37 ± 1.07**	10.2
GluAp (mU/mg protein)	2.95 ± 0.68	12.2 ± 0.67**	4.13
*Exosomal fraction*			
GluAp (mU/ml)	0.10 ± 0.03	0.38 ± 0.04	3.70
GluAp (mU/mg creatinine)	0.19 ± 0.05	3.34 ± 0.59**	17.6
GluAp (mU/100g/day)	0.40 ± 0.12	3.81 ± 0.50**	9.45
GluAp (mU/mg protein)	1.19 ± 0.34	7.01 ± 0.55**	5.88

GluAp activity in supernatant, microvesicular and exosomal fractions of urine samples from Control and Cisplatin groups collected 3 days after injection. Data were expressed in mU/ml, mU/mg creatinine, mU/100g/day and mU/mg protein. Mean ± SEM

* p<0.01

** p<0.001 Cisplatin *vs*. Control (n = 10 each group).

Interestingly, in this experiment, urinary creatinine excretion was decreased in cisplatin-treated group because of decreased glomerular filtration (Table 5). Therefore, in this situation, normalization of urinary markers by creatinine would falsely increase their urinary excretion. Note that the relative increase of GluAp per mg of creatinine is always higher than the relative increase of the enzyme when activity is normalized by body weight or total protein content of each fraction (Table 4). This finding remarks the needing of methods of quantification for spot samples other than normalization by creatinine concentration in order to study renal alterations of nephron segments independently of glomerular filtration status. In our study, we found a high excretion of GluAp per mg of protein in microvesicular and exosomal fractions that could be used to evaluate the extent of proximal tubular toxicity after cisplatin injection.

**Table 5:** Urinary variables 3 days after injection.

	Control	Cisplatin
UCr (mg/100g/day)	2.14 ± 0.11	1.23 ± 0.15**
Proteinuria (mg/mg crea)	0.82 ± 0.03	7.31 ± 0.72**
Microvesicular protein (μg/mg crea)	145 ± 9.34	705 ± 118**
Exosomal protein (μg/mg crea)	168 ± 16.8	491 ± 88.5**
Diuresis (ml/100g)	3.95 ± 0.47	10.3 ± 1.36*

Urine creatinine excretion (UCr; mg/100g/day), proteinuria (mg/mg creatinine), microvesicular (μg/mg creatinine) and exosomal (μg/mg creatinine) protein content, and diuresis (ml/100g) in Control and Cisplatin groups 24 hours after injection. Data are expressed as mean ± SEM.

* p<0.01

** p<0.05 Cisplatin *vs* Control (n = 10 each group).

Furthermore, cisplatin-treated rats displayed high proteinuria at this point (Table 5), and quantification of GluAp in supernatant fraction was falsely decreased when divided by supernatant protein.

It is also remarkable that GluAp remained increased in microvesicular and exosomal fractions of cisplatin-treated rats, although protein content was higher in both fractions in this group (Table 5).

AlaAp activity, which has been described as an exosomal marker of proximal tubular damage [11, 16] showed a very similar pattern to GluAp in Cisplatin group (Table 6).

**Table 6:** AlaAp activity in urine fractions.

	Control	Cisplatin	Relative increase
*Supernatant*			
AlaAp (mU/ml)	1.20 ± 0.11	4.04 ± 0.40**	3.36
AlaAp (mU/mg creatinine)	2.27 ± 0.33	34.1 ± 4.77**	15.0
AlaAp (mU/100g/day)	4.96 ± 0.87	41.1 ± 7.31**	8.29
AlaAp (mU/mg protein)	2.78 ± 0.40	4.67 ± 0.46*	1.68
*Microsomal fraction*			
AlaAp (mU/ml)	0.66 ± 0.05	1.83 ± 0.12**	2.79
AlaAp (mU/mg creatinine)	1.18 ± 0.11	16.1 ± 2.34**	13.6
AlaAp (mU/100g/day)	2.57 ± 0.32	19.0 ± 2.92**	7.40
AlaAp (mU/mg protein)	8.39 ± 1.02	23.8 ± 1.59**	2.83
*Exosomal fraction*			
AlaAp (mU/ml)	0.44 ± 0.05	0.75 ± 0.08*	1.72
AlaAp (mU/mg creatinine)	0.78 ± 0.09	6.50 ± 1.07**	8.37
AlaAp (mU/100g/day)	1.64 ± 0.20	7.84 ± 1.46**	4.78
AlaAp (mU/mg protein)	4.88 ± 0.63	14.6 ± 2.18	2.99

AlaAp activity in supernatant, microvesicular and exosomal fractions of urine samples from Control and Cisplatin groups collected 3 days after injection. Data were expressed in mU/ml, mU/mg creatinine, mU/100g/day and mU/mg protein. Mean ± SEM

* p<0.01

** p<0.001 Cisplatin *vs*. Control (n = 10 each group).

**Fig 6:**
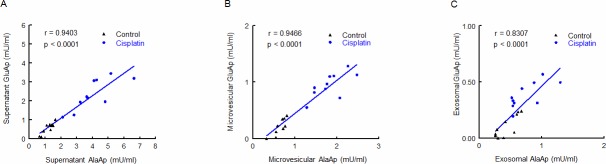
Correlations between GluAp and AlaAp. Linear regressions between GluAp activity (mU/ml) in supernatant (A), microvesicular (B) and exosomal (C) fraction of urine samples collected 3 days after injection.

AlaAp and GluAp activities were highly correlated in supernatant, microvesicular and exosomal fractions (Fig 6). These data clearly suggest that GluAp must also be considered as a marker of proximal tubular toxicity in these fractions.

### GluAp activity were highly correlated with SCr at the peak of toxicity

**Fig 7:**
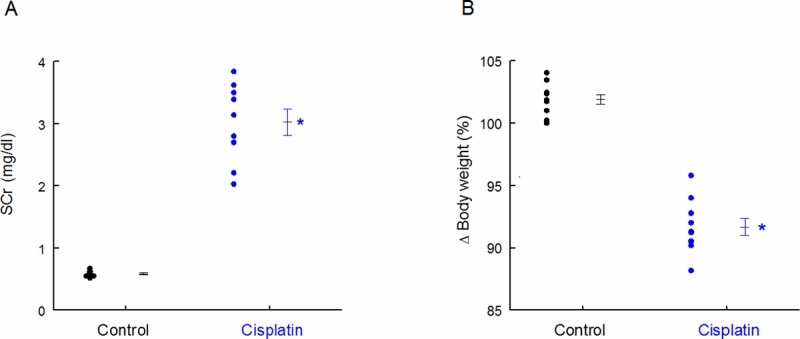
SCr and body weight. SCr concentration (mg/dl) (A) and body weight increase (%) in Control and Cisplatin groups 3 days after injection. Individual samples and means ± SEM are displayed; * p<0.001 Cisplatin *vs*. Control (n = 9–10 each group).

Three days after injection, SCr was very increased and body weight was decreased (Fig 7), probably due to volume loss because of high diuresis. Correlation coefficients and p-values of GluAp and AlaAp in the different fractions with SCr at this point are shown in Table 7 and Table 8, respectively. These correlations were stronger for GluAp in all studied fractions, and they were also stronger than correlations determined in the first experiment. The lowest correlations were found when data were normalized by total protein in supernatant fraction, further demonstrating that determinations of GluAp in microvesicular and exosomal fractions can be of interest to evaluate proximal tubular damage.

**Table 7:** Correlations between GluAp activity and SCr.

	r	p
*Supernatant*		
GluAp (mU/ml)	0.8597	<0.001
GluAp (mU/mg creatinine)	0.8832	<0.001
GluAp (mU/100g/day)	0.7683	0.0001
GluAp (mU/mg protein)	0.6138	0.0052
*Microsomal fraction*		
GluAp (mU/ml)	0.9115	<0.0001
GluAp (mU/mg creatinine)	0.8007	<0.0001
GluAp (mU/100g/day)	0.7986	<0.0001
GluAp (mU/mg protein)	0.8599	<0.0001
*Exosomal fraction*		
GluAp (mU/ml)	0.8217	<0.0001
GluAp (mU/mg creatinine)	0.7673	0.0001
GluAp (mU/100g/day)	0.7605	0.0002
GluAp (mU/mg protein)	0.7897	0.0001

Correlation coefficient (r) and statistical significance (p) of linear regressions between GluAp activity expressed in mU/ml, mU/mg creatinine, mU/100 g/day and mU/mg protein in supernatant, microsomal and exosomal fraction of urine samples collected 3 days after injection, with SCr concentration determined at this point.

**Table 8:** Correlations between AlaAp activity and SCr.

	r	p
*Supernatant*		
AlaAp (mU/ml)	0.8350	<0.0001
AlaAp (mU/mg creatinine)	0.7935	0.0001
AlaAp (mU/100g/day)	0.6348	0.0035
AlaAp (mU/mg protein)	0.4732	0.0407
*Microsomal fraction*		
AlaAp (mU/ml)	0.8881	<0.0001
AlaAp (mU/mg creatinine)	0.7793	0.0001
AlaAp (mU/100g/day)	0.6702	0.0017
AlaAp (mU/mg protein)	0.7717	0.0001
*Exosomal fraction*		
AlaAp (mU/ml)	0.6027	0.0063
AlaAp (mU/mg creatinine)	0.7385	0.0003
AlaAp (mU/100g/day)	0.5865	0.0083
AlaAp (mU/mg protein)	0.5467	0.0154

Correlation coefficient (r) and statistical significance (p) of linear regressions between AlaAp activity expressed in mU/ml, mU/mg creatinine, mU/100 g/day and mU/mg protein in supernatant, microsomal and exosomal fraction of urine samples collected 3 days after injection, with SCr determined at this point.

Exosomal fraction has been widely used for biomarkers research studies, and there are evidences of physiological and pathological roles of exosomes in the kidney, where regulate the co-functioning between different parts of the nephron, through secretion and reuptake of their contents such as mRNAs and miRNAs that can affect the function of the recipient cell [14,21]. Nevertheless, microvesicles have not received enough attention in biomarker research, although their secretion has been studied in some renal pathologies [22,23], and they also contain mRNA and miRNA that might play a role in transferring information in the kidney [24,25]. Obtention of microsomal fraction is easier and faster than exosomal fraction, and our results demonstrate that it can also constitute an important source of biomarkers related to renal dysfunction. The presence of GluAp and AlaAp in microvesicles, which has not been described in previous bibliography, can be related with the fact that microvesicles are originated from direct budding of tubular cell membranes [22] that contain aminopeptidasic enzymes [19], and it cannot be discarded that large membrane fragments could also contribute to the increased enzymatic activities that we have found in this fraction.

### Expression of GluAp, Alix and TSG101 in microvesicular and exosomal fractions

**Fig 8:**
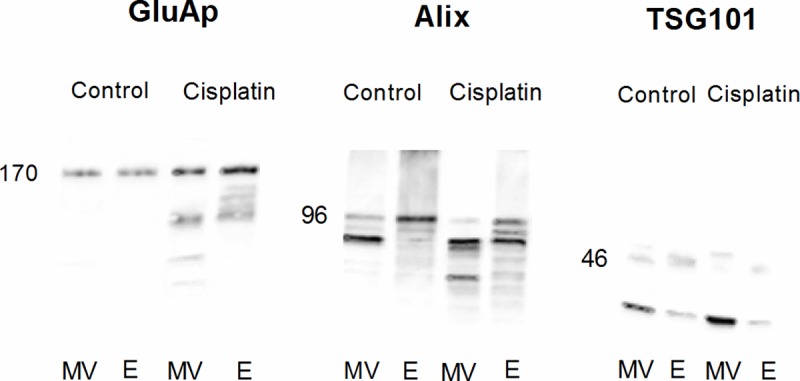
Immunoblotting of GluAp, Alix and TSG101. Expression of GluAp, Alix and TSG101 in microvesicular (MV) and exosomal (E) fractions from urines collected 3 days after treatment in Control and Cisplatin groups. Each lane contains 30 μg of protein.

Immunoblotting also demonstrated that GluAp was increased per mg of protein in microvesicular and exosomal fractions of urine from cisplatin-treated rats (Fig 8, left).

In order to test the enrichment in extracellular vesicles of these fractions, we analyzed the expression of Alix and TSG101, accesory proteins that are required for the formation of multivesicular bodies [26] and are present in exosomes irrespectively of cell type [27]. TSG101 has also been described in microvesicles where is involved in direct membrane budding [28].

In our study, we found a higher expression of Alix at 96 kDa (Fig 8, middle) and TSG101 at 46 kDa (Fig 8, right) in exosomal fractions than in microvesicular fractions from both control and cisplatin group, confirming the presence and enrichment of exosomes in this fraction.

Interestingly, both TSG101 and Alix were also detected in microvesicular fraction, but the pattern of expression was completely different to that of exosomal fraction. These differences in electrophoretic migration of both proteins can be due to the fact that microvesicles carry more proteins with posttranslational modifications when compared with exosomes [29]. In the case of TSG101, there was even an accessory band at 25 kDa that was very patent in microvesicular fraction from both control and cisplatin groups, but it was slightly detected in exosomal fraction.

It is also remarkable that the expression of Alix and TSG101 was very similar in cisplatin than in control group, and GluAp was the only marker that was clearly overexpressed in cisplatin-treated rats in both fractions, strengthening the role of the content of GluAp in these fractions as a marker of nephrotoxicity.

## Conclusions

GluAp content or its enzymatic activity in microvesicular and exosomal fractions of urine has been shown to be an early and predictive biomarker of renal dysfunction in cisplatin-induced nephrotoxicity.

These variables are useful to evaluate proximal tubular damage regardless of glomerular filtration rate.

## Supporting information

S1 File Individual data. Individual data of body weight, diuresis and biochemical measurements in samples collected 24 hours (Experiment 1) and 3 days (Experiment 2) after cisplatin injection.
(XLS)
